# WELCOME: improving WEight controL and CO-Morbidities in children with obesity via Executive function training: study protocol for a randomized controlled trial

**DOI:** 10.1186/s12889-018-5950-3

**Published:** 2018-08-29

**Authors:** Tiffany Naets, Leentje Vervoort, Marijke Ysebaert, Annelies Van Eyck, Stijn Verhulst, Luc Bruyndonckx, Benedicte De Winter, Kim Van Hoorenbeeck, Ann Tanghe, Caroline Braet

**Affiliations:** 1 Department of Developmental, Personality and Social Psychology, Ghent University, Henri Dunantlaan 2, 9000 Ghent, Belgium; 2 Department of Pediatrics, Antwerp University and University Hospital Antwerp, Antwerp, Belgium; 3 Zeepreventorium, vzw, De Haan, Belgium

## Abstract

**Background:**

Obesity is a widespread problem that not only leads to medical and psychological diseases in adults, but also in children and adolescents at an early stage in life. Because of its global burden on both the individual and society, it is necessary to develop effective evidence-based treatments. Current “Multidisciplinary Obesity Treatments” (MOT) already provide significant weight loss, but still leave room for more long-lasting improvements. In this protocol paper, we outline the research goals of the WELCOME trial, based on a substantial proof of concept.

**Methods:**

In this Randomized Controlled Trial (RCT) – conducted in both an inpatient and two outpatient treatment settings – existing MOT will be supplemented with an Executive Function (EF) training and compare effects on various parameters in an experimental versus an active control group of obese youngsters (8–18 years old). WELCOME aims to (a) train youngsters’ executive functions to facilitate effects on weight loss, psychological and medical comorbidities, (b) to enhance the long-term effects by continuing the training in the daily home context with booster sessions, and (c) to investigate its effects until a 6-month follow-up. In comparison to the active control group, better progress is expected in the experimental group on following variables: weight, psychological comorbidities (unhealthy eating behavior, internalizing symptoms, impaired self-esteem) and medical comorbidities (metabolic syndromes, endothelia dysfunction, tonsillar hypertrophy and sleep obstruction).

**Discussion:**

It is stated that this EF-training for enhancing self-control abilities is necessary for a long-lasting effect of childhood obesity treatment interventions.

**Trial registration:**

The Study Procotol was registered on 10/05/2017 (n° ISRCTN14722584).

## Background

The prevalence of childhood obesity has reached the level of an epidemic, with currently 42 million children worldwide being overweight or obese [1]. Chronic weight problems pose serious medical and psychosocial health risks, including metabolic syndromes, cardiovascular diseases, sleep problems, depression, anxiety and decreased self-esteem [2–5]. These problems already develop at an early age and often continue into adulthood, with 60% of the individuals who were overweight at 11 years old growing up to be overweight adults [6].

Tackling weight problems in children is undeniably one of the most promising strategies to decrease the impact of obesity. The current golden standard for treating childhood obesity is an evidence-based Multidisciplinary Obesity Treatment (MOT): a family-based intervention that not only focuses on changes in diet and physical activity (to reduce the imbalance between energy-intake and energy-expenditure), but that also pays attention to healthy lifestyle behavior and psychosocial wellbeing [7, 8]. Evidence shows that MOTs have a clinically significant impact on the weight, physical fitness and psychosocial wellbeing of young children as well as adolescents [9–11]. However, research also shows that existing treatments only have limited long-term success. Especially weight control after MOT appears to be difficult, leading to weight regain at follow-up [7, 11, 12]. It is therefore crucial to keep up the efforts to optimize existing interventions for obese children, and to maximize their long-term effects.

Currently, research emerges on self-control deficits in obese individuals to explain these findings, and – even more importantly – to lead to new treatment options [13]. Self-control implies “Executive Functioning” (EF): the neuropsychological system that underlies self-control by activating and regulating goal-directed behavior in response to the environment [14, 15]. EF is involved in high-level tasks such as planning, organization, etc., but also in the basic cognitive processes responsible for guiding behavior [16]. Problems in EF are shown to be involved in weight problems, with poor EF hindering the development of skills to resist food temptation, and predicting weight gain from pre-school to adulthood ([17–21]). Moreover, EF-problems are also observed in weight related medical comorbidities such as the Obstructive Sleep Apnea Syndrome (OSAS), oxidative stress and subclinical inflammation [5], and in the psychological comorbidities such as depression or other internalizing problems ([22, 23]). Furthermore, EF-deficits also affect treatment: children and adults who have EF-problems experience more difficulties in losing weight both at a short and long term, and also prematurely drop out of treatment more often ([24–28]). The influence of underlying EF-mechanisms could therefore at least partially explain the rather modest long-term results of existing MOT.

More specifically, two EF-abilities are of crucial importance: inhibitory control and attentional processing. The Dual Pathway Model of Appelhans et al. [29] describes the associations between inhibition, attention and obesity. The model states that neuropsychological self-control behavior is generated by both top-down and bottom-up processes as two interactive pathways ([16, 29]). Bottom-up, there is a fast and automatic system that reacts to stimuli based on their emotional and motivational value, and directs *attention* towards them. Top-down processes are needed to regulate these automatic reactions to reach self-control. They are part of a slow, reflective system that can consciously *inhibit* responses by deliberation [29]. Both bottom-up and top down processes are impaired in obesity. Obese individuals are excessively reactive to high-caloric food stimuli, via an attentional bias towards them [30]. Furthermore, they have difficulties suppressing automatic approach behaviors towards food, compared to non-overweight individuals [31]. When individuals both have excessive attentional bottom-up reactivity and impaired top-down inhibitory control, they are at the highest risk for developing long-lasting weight problems [29].

Fortunately, innovative research exploring the potential of strengthening self-control through computerized training of attention and inhibition, shows that EF-training can improve self-control and even lead to weight decrease. Inhibition training – aimed at triggering stop-reactions instead of approach behavior when confronted with unhealthy food stimuli – can have a beneficial effect via food intake and sustained weight loss in overweight and obese adults, up until 6 months after training [32, 33]. Research on children is rather scarce, but the existing evidence in this age group is also promising [13, 34]. For example, in the study of Folkvord et al. [34], overweight children completed an inhibition game and showed a decrease in caloric intake already after one session. Furthermore, also attentional biases can be modified in both obese adults and children, redirecting attention away from (unhealthy) food [35–38], with corresponding changes in eating-related variables and weight, up until three months’ follow-up [35]. For example, the Attention Modification Program in obese children resulted in decreased caloric intake [36]. It is therefore meaningful to further explore the possibilities of such trainings for the improvement of obesity treatments.

Although results are promising, there are still several research gaps that have to be taken into consideration. For example, merely a few EF-trainings, and only in adults, combine top-down inhibition and bottom-up attention as processes following the comprehensive model of Appelhans (2011) and colleagues [32]. The most challenging innovation is to investigate if these training effects are consistent in children, especially on a long-term. So far, only the study of Verbeken et al. [13] proved their 6-week EF-training to be effective, although the effects of their training on BMI disappeared after 12 weeks. It is possible that the study’s 6-week training phase was too short in timespan and lacked intensity to produce long-lasting training effects. For this reason, it is useful to investigate the possibilities of homework booster sessions. By extending the learning possibilities over a longer term, the child can implicitly incorporate the acquired skills into daily routines in various situations, which might result in enhanced self-control necessary for maintaining a healthy lifestyle [13].

In sum, based on the promising findings of EF-training together with its challenges, the WELCOME-trial (“*Improving WEight ControL and CO-Morbidities in children with obesity via Executive function training*”) has been developed. In this project, a new EF-training will be evaluated via a Randomized Controlled Trial in both inpatient and outpatient treatment settings. The training will be implemented on top of existing MOT’s, because EF-training alone cannot replace existing evidence-based treatments [12]. The EF-training is unique as it based on the dual-process model of Appelhans (2009), combining a top-down inhibition and a bottom-up attention training, executed for 6 weeks in an intensive phase during MOT (twice a week). Based on the evaluation of Verbeken, et al. [13] the EF training will be continued for 8 weeks in a booster phase directly after the intensive phase of the MOT (once a week). It is expected that this EF-training will lead to better effects, in comparison to an active control group that will receive a sham-training during the same period with the same components (inhibition and attention) but without active learning ingredients. More exactly, it is expected to find enhanced outcomes in the experimental group on several parameters: (a) more weight loss, (b) an improvement on executive functions, (c) reduced psychological comorbidities such as decreased maladaptive eating behavior, less internalizing problems and a higher self-esteem, and (d) a decrease in problematic medical parameters such as metabolic syndromes, tonsillar hypertrophy, endothelia dysfunction and sleep obstruction. Furthermore, sustainability of the training effects will be evaluated at long term (6 months follow-up). The protocol was registered at ISRCTN Registry, number 14722584.

## Methods

### Study design and randomization

The effectivity of a computerized “Brain Fitness” EF-training program will be investigated in a prospective, multi-centered RCT by comparing an experimental training group to an active control group in three treatment centers: one inpatient facility (*Zeepreventorium vzw*, De Haan, Belgium) and two outpatient centers (*Jan Palfijn Ziekenhuis Ghent, Belgium* and *Universitair Ziekenhuis Antwerpen*, Belgium). Full information on these participating centers is listed in the Clinical Trials Register with ISRCTN 14722584. The study will be blinded to both participants and treatment center staff (including therapists and laboratory personnel).

Assessment will be done at **five** time points: (1) at admission of the MOT – Multidisciplinary Obesity Treatment (**T0**, pre-treatment), (2) 6 weeks before the end of the intensive period of MOT (approximately 4–10 months after start of treatment) (**T1**, pre EF-training), (3) at the end of the intensive phase of both the MOT and the EF-training on top of the ongoing MOT (**T2**, post EF-training), (4) the end of the booster EF-training with post-treatment personalized contacts in the MOT (**T3**) and (5) a 6-month follow-up after the end of the intensive MOT phase (**T4,** follow-up). For an overview, we refer to the SPIRIT schedule (Table 1; [39]).

**Table 1:**
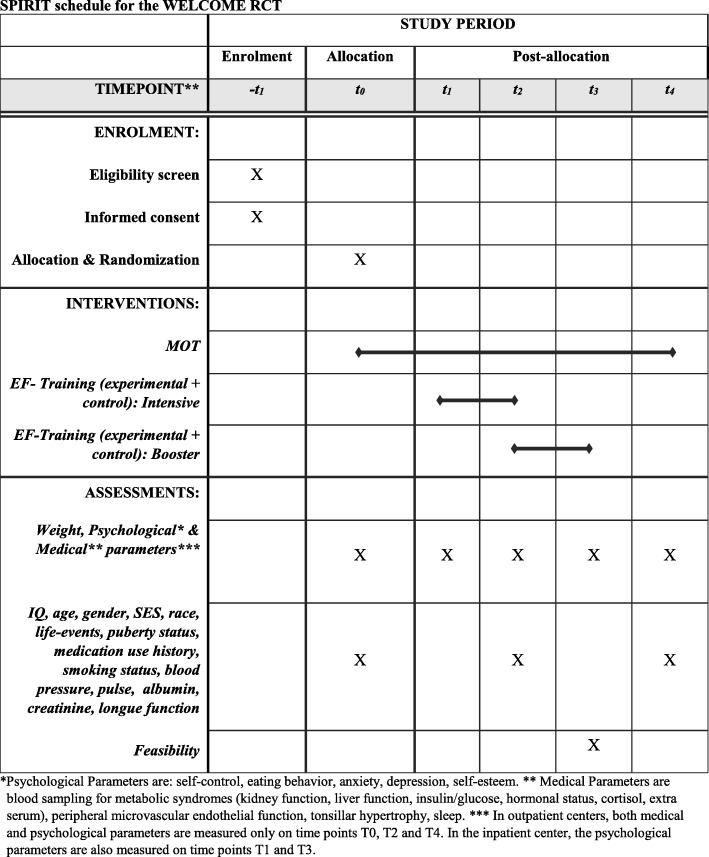
SPIRIT schedule for the WELCOME RCT

^a^Psychological Parameters are: self-control, eating behavior, anxiety, depression, self-esteem

^b^Medical Parameters are blood sampling for metabolic syndromes (kidney function, liver function, insulin/glucose, hormonal status, cortisol, extra serum), peripheral microvascular endothelial function, tonsillar hypertrophy, sleep

^c^In outpatient centers, both medical and psychological parameters are measured only on time points T0, T2 and T4. In the inpatient center, the psychological parameters are also measured on time points T1 and T3

### Recruitment & eligibility

The 2-year recruitment of participants for this study started in 2017. Possible participants are informed at intake (T0) and an active written informed consent from both children and parents or legal guardians is required for inclusion. All data collection procedures are approved by the Institutional Ethical Committee and have been performed in accordance with the ethical standards laid down in national laws and in the 1964 Declaration of Helsinki and its later amendments.

The inclusion criteria of the study are: (a) age 8–18 at intake (T0) and (b) presence of overweight or obesity with a cut-off BMI, indexed with IOTF cut-off and BMI z-scores based on Flemish growth curves (compared to an age and gender appropriate norm group; [40]). Exclusion criteria are: (a) participation in another intervention trial and (b) medical problems such as inflammatory diseases, structural heart or other cardiac diseases, active malignant hematological illness, neurological condition and obesity caused by a genetic or endogenous disease such as central hypothyroidism.

### Measurements

#### Body weight

Participants’ weight will be indexed by the Body Mass Index (BMI), with the BMI-SDS and IOTF cut-off as most crucial weight indexes for children and adolescents [41]. First, the BMI standard score is calculated by subtracting the mean from the individual BMI weight score and dividing that number by the standard deviation, based on normative data in an age and gender dependent (Flemish) sample [42]. Second, the International Obesity Task Force (IOTF) published cut-off criteria for childhood overweight (25 kg/m^2^) and obesity (30 kg/m^2^), based on the centiles on age and gender specific growth charts [40].

#### Executive functions

To evaluate EF as specific self-control abilities, we will use both task and questionnaire data.

##### Inhibition: go/no-go

A food-specific Go/No-Go [43] will be used to measure top-down inhibitory control. In each trial, a neutral or unhealthy stimulus appears on the screen, simultaneously with a letter (“q” or “p”) for a maximum period of 1500 ms. Participants are instructed to respond (pressing the spacebar) as quickly as possible when the picture is accompanied by a go-stimulus (e.g. “p”; go-trial) or to do nothing when they see a no-go stimulus (e.g. “q”; no-go-trial). The cues (action with “p” or “q”) and the response assignment (go versus no-go) are counterbalanced, and the presentation of the pictures differs randomly for each participant. There are pictures of 8 unhealthy and 8 neutral stimuli in two blocks of 160 trials, preceded by a practice phase with 8 animal pictures. The outcome variables are reaction time, commission error (errors representing failed inhibition) and omission errors (errors representing not reacting when supposed to).

##### Attention: dot probe

This paradigm was adapted from MacLeod, Mathews and Tata (1986) to a food-specific bottom-up attention bias module. First, a white cross is presented in the middle of the screen for 500 ms. Next, also for 500 ms, two pictures are presented left and right on the screen (an unhealthy and a neutral photo) with similar characteristics (same color, size, etc.). After the pictures disappear, a white dot replaces one of the pictures. The instruction here is to press “e” on a keyboard when the dot stands on the left, and “i” when it stands on the right side of the screen, randomly presented for each participant. There are 16 unhealthy-neutral pairs of stimuli with two blocks of 130 trials. The outcome variable is reaction time.

##### Visual search task

To measure transfer effects to other known EF-tasks, we will also include a measurement of EF with an adapted “odd-one-out” visual search task [44–46]. Participants have to indicate the location of a stimulus that does not fit into the category of the 5 × 5 matrix (“odd-one-out stimulus”) as quickly as possible. Participants will search for food pictures among non-food pictures and vice versa, and their response times will be measured to compare speed of detection between both groups. Within the trials, there is a fixation time for 500 ms, followed by the matrix that shifts after the response of the participant with inter-trial intervals of 1000 ms. After each trial, feedback will be provided.

##### EF-questionnaires

Self-reported and parent- or educator reported EF will be assessed with the Behavior Rating Inventory of Executive Functioning (BRIEF, [47]). The BRIEF contains 75 items on a 3-point Likert scale, ranging from “never” to “often” applicable to the youngster, referring to eight subscales: inhibition, flexibility, emotional control, initiation, working memory, planning/organizing, and monitoring. Related to EF, we also include the Effortful Control Scale (ECS, [48]), representing basic self-control abilities [16]. The ECS contains 24 items that need to be answered on a five-point Likert scale (ranging from “not at all” to “completely suitable”). For both instruments, a higher score represents more self-control problems. Psychometric properties of both instruments are good to strong [49, 50].

### Psychological comorbidities

#### Maladaptive eating behaviors

Two self-report measurements will be used to measure unhealthy eating behavior: the Dutch Eating Behavior Questionnaire (DEBQ, [51]) and the Children’s Eating Disorder Examination interview – Questionnaire (Ch-EDE-Q, [52, 53]). The DEBQ contains 33 items on a 5-point Likert scale (ranging from never to very often), relating to three subscales: emotional, external and restrained eating. The Ch-EDE-Q contains 30 items on a 7-point Likert-scale (ranging from 0 to 28 days present in the last month); in which we are mainly focused on the three questions on binge eating. Higher scores reflect more maladaptive eating behavior in the Ch-EDE-Q and DEBQ. Both instruments are frequently used for screening eating pathologies and have good psychometric properties [54–56].

#### Internalizing problems

Self-report and parent-report measurements will be used to assess internalizing problems such as self-esteem issues, anxiety and depression. The Dutch child and parent version of the Achenbach System of Empirically Based Assessment (ASEBA, Youth-Self-Report YSR and Child Behavior Checklist CBCL; [57, 58] contain 113 items on a 3-point Likert scale ranging from never to often, and represent three global scales (internalizing, externalizing and total problem behaviors) next to several behavioral and DSM-IV oriented subscales. Next, the translated self-report Harter’s Self Perception Scale [59] contains 35 items on a 4-point scale that refer to self-esteem on seven life domains: school, social acceptance, sports, physical appearance, behavioral conduct, friendship and global self-esteem. On the ASEBA, higher scores reflect more problems. On the Harter scales, higher scores indicate higher self-esteem. Both instruments have good psychometric properties and are frequently used both in research and clinical contexts [60, 61]).

### Medical comorbidities

#### Metabolic syndrome

Via blood sampling, overnight fasting will be obtained from an antecubital vein. Serum will be centrifuged after blood collection and aliquots stored at − 80 °C until analysis. Routine blood parameters will be analyzed at certified laboratories (Zeepreventorium or UZA). Fasting blood samples are analyzed immediately for levels of glucose, insulin, total cholesterol, triglycerides, LDL and HDL cholesterol and hs-CRP. The following analyses will be performed on stored serum/plasma samples: total adiponectin levels (Human Adiponectin Quantikine ELISA, R&D) leptin levels (Human Leptin Quantikine ELISA, R&D).

#### Endothelial dysfunction

Peripheral microvascular endothelial function can be noninvasively measured at the distal phalanx of the index fingers with Endo-PAT (Itamar Medical Ltd). This involves a plethysmographic evaluation of small arterial pulsatile changes on the fingertip at rest and during reactive hyperaemia after 5 min. of forearm ischemia by means of inflating and deflating a forearm blood pressure cuff. Measurements will be done following recommendations of Bruyndonckx et al. [3].

#### Tonsillar hypertrophy

The degree of tonsillar hypertrophy will be clinically assessed by using the Brodsky score for tonsillar hypertrophy, which ranges from a value of 0–4+. Tonsillar hypertrophy will be defined by a Brodsky score of 3+ or 4 + .

#### Sleep problems

A portable device (ApneaLink, ResMed; [62–64]) will be used to perform a sleep assessment when SDB was observed during the first investigation. Respiratory airflow will be measured by a nasal pressure cannula (detecting − 10 hPa to + 10 Hpa) and oxygen saturation (Sa02), and pulse rate will be obtained by using both a pulse oximeter and a pule sensor (sampling rate of 1 Hz). Apnea is defined as the absence of airflow lasting at least two breaths. Hypopnea is a ≥ 50% decrease in amplitude of the airflow signal, lasting for ≥2 breaths and with a concurrent desaturation of ≥4%. The respiratory disturbance index (RDI) is calculated as the sum of the recorded apneas and hypopneas divided by the total sleep time. Since ApneaLink is a screening device, it does not equal full polysomnography. Therefore, it is not possible to make the difference between obstructive and central events, nor to recognize arousals. All desaturations of ≥4% from the baseline SaO2 will be quantified and the oxygen desaturation index (ODI) will be calculated as the total of desaturations divided by the total sleep time. The mean nocturnal SaO2 (<SaO2>) and SaO2 nadir will be registered. Since ApneaLink does not differentiate between central or obstructive respiratory events, mild SDB will be diagnosed in the presence of an ODI between 2 and 5 and severe SDB will be defined by an ODI of ≥5 [62, 64–66].

### Description of the involved interventions

#### Multidisciplinary obesity treatment (MOT)

Each participant in this study will receive a treatment program based on an evidence-based MOT protocol.

#### Inpatient treatment at the Zeepreventorium VZW

This treatment consists of a 10-month evidence-based MOT [67] for severely obese children and adolescents. A healthy body weight is the primary aim, throughout teaching healthy food behavior and daily physical activity, using various multidisciplinary interventions such as Cognitive Behavioral Techniques (CBT). The parents are involved at a frequent base.

#### Outpatient treatment at the Antwerp University Hospital

A standardized outpatient MOT for obese children and adolescents of 12–24 weeks, is a problem-oriented approach in dialogue with patients and their parents. It starts with a hospitalization to objectify the severity of the obesity and comorbidities. A multidisciplinary team, supervised by a pediatrician, then monitors general progress in eating behavior, physical activity, and comorbidities. Patients are seen biweekly at the intensive phase of 12 weeks, focusing on a healthy diet in a step by step method according to a documented protocol.

#### Outpatient treatment at the Jan Palfijn Hospital

An evidence-based program focuses on three components within behavioral lifestyle: healthy eating habits, moderate exercise and Cognitive Behavioral Therapy. A multidisciplinary team, also supervised by a pediatrician, monitors the patient every 2 weeks for an intensive phase of 12 weeks. The standardized outpatient MOT protocol is evidence based, and described in detail in Braet & Van Winckel [7]. The parents are involved at a frequent base.

#### Intervention: EF-training (“brain fitness”)

In addition to the MOT, each participant will also receive a computerized EF-training in two stages. First, they will train EF abilities during the MOT-intensive phase; containing 12 sessions of approximately 30 min, twice a week for 6 weeks. Second, there is a booster phase directly after completion of the intensive MOT phase, with 8 sessions during 8 weeks (once every week). This training is web-based, so this can be executed in the treatment facility as well as at home. This web-based format allows to track the evolution of each participant, and to send them an invitation each time they have to train.

The experimental as well as the control (sham) training consists of two food-specific tasks targeting inhibition and attention:

#### Inhibition training: go/no-go

The goal of is to systematically learn to suppress a response towards unhealthy food stimuli. The training manipulation is based on a 90/10 contingency relationship between the letters and the stimuli. For the experimental group, there is a connection between the cue (no/go) and the nature of the picture (healthy/neutral). In 90% of the cases, the no-go cue is paired with an unhealthy picture, expected to lead to more inhibition towards unhealthy food [43]. In the sham-training of the active control group, the only difference is a 50/50 contingency relationship between the cues and the stimuli [32].

#### Attention training: dot probe

The goal of this task is to transform an attention bias towards unhealthy food into a bias away from unhealthy food. Again, in the training manipulation, there exists a 90/10 a contingency relationship between the location of the dot (left-right) and the nature of the picture (unhealthy/neutral). In 90% of the trials, the dot appears on the location of the cue with a neutral photo, to direct attention away from unhealthy food. For the sham-training in the active control group, the only difference is the equal 50/50 proportion in dots paired with unhealthy and with neutral photos [68].

#### Drop-out, motivation and feasibility

Drop-out is one of the biggest challenges in treatment, and it is often an important explanation for non-optimal effectiveness [69]. The drop-out rates in obese youth during MOT are rather high, sometimes around 27% in hospital-based clinics [70]. It is therefore important to prevent attrition at an early stage, by increasing motivation to complete the training. In this study, we will add several motivational components that are assumed to enhance participation because of their high rewarding value: providing insight in the rationale of the training with attractive materials (such as a “Brain Fitness Guide”) for both children and their parents, giving a scoring schedule that allows participants to save up for a reward, guaranteeing variation in the training sessions (by combining two tasks (attention and inhibition) and by administering different stimuli sets), the use of personalized e-mail reminders, and the use of limited game elements by animated compliments after each session. Furthermore, we will also evaluate the feasibility of the training with participants, their parents and the MOT care providers. Those insights will be supplemented with the drop-out rates and cost and time efficiency analyses. Both insights on motivational encouragement and feasibility analysis could generate a better understanding of drop-out and lead to more satisfaction for users and better adherence rates [71].

### Aims & hypotheses

This RCT mainly aims to test if the computerized program – with 6 weeks intensive and 8 weeks booster training, with a total of 20 training sessions of self-regulation training focusing on inhibition and attention on top of MOT – helps obese children lose weight and maintaining weight loss. We expect that the experimental group will have lost more weight in comparison to the active control group.

To gain insight in the mechanisms of such treatment effect, we also aim to investigate whether or not the training indeed improves self-control and whether these improvements contribute to weight loss (changes in self-control as proposed mediator of treatment effect). Increased self-control is expected in the experimental group compared to the control group, both on the self-control questionnaires and the inhibition and attention task. Additionally, it is useful to investigate transfer of learning by testing performance in the visual search task [44] which comprises both attention and inhibition.

Next to the effects of the primary outcome “weight” (as indexed by a.05 BMI-SDS decrease), more beneficial effects of the training are expected in the experimental group compared to the control group on secondary outcomes: (a) a decrease in maladaptive eating behavior expressed in lower scores on binge eating (Ch-EDE-Q), external eating (DEBQ) and emotional eating (DEBQ); (b) a decrease in comorbid internalizing behaviors expressed in a lower score on internalizing problems (CBCL, YSR) and in a higher feeling of self-esteem (CBSK and CBSA) and (c) a decrease in the components of the metabolic syndrome, endothelial function and OSA. Furthermore, we will explore the influence of potential moderators of the treatment effect: age, gender, IQ, initial BMI-SDS and presence of OSA.

### Statistical analysis

#### Sample size

Power calculations (G*-power, [72]) showed that at least 200 participants (90 per group with 10% drop-out) are needed to detect an effect size of *f* = .10 [73, 74] on weight as the primary outcome (power of .8 & significance level of.05). In the inpatient center, 100 participants will be recruited, and 50 in both outpatient centers.

#### Analytic plan

All data will be analyzed via the intention-to-treat (ITT) principle [75]. Treatment effects will be tested using multilevel analyses, allowing to handle missing and nested data from the three treatment facilities [76]. An autoregressive type of repeated covariance and independent variables will be set up in a model with “group” (experimental, control) and “time” (T0-T4), all two-sided on a *p*-value of <.05 and with effect sizes of generalized eta squares. Data analyses will be documented in a data management form (i.e. about version tracking, save locations, etc.) for safe storage, secrecy and data security.

## Discussion

Childhood obesity is a widespread problem in most Western countries, especially since it is accompanied by large negative medical, psychological, social, and economic consequences that continue into adulthood when untreated [2, 3, 77]. Multidisciplinary Obesity Treatments (MOT) – combining diet, behavioral change, and physical activity – are considered to be effective [10]. However, there is still room for improvement, since their long term effects are quite small and weight re-gain is often observed [12]. The scientific community therefore has an important responsibility to develop and study interventions aimed at sustainable long-term results, that also tackle underlying mechanisms of change [1].

Deficits in executive functioning can be a possible explanatory mechanism for the modest results of treatments, given the omnipresent temptations of unhealthy food in our current obesogenic environment [78]. Obese individuals (both youth as adults) show marked EF-deficits, including impaired top-down inhibition and food-related bottom-up attentional bias [13, 29, 79, 80]. Fortunately, there is preliminary evidence for the malleability of these processes in both adults and youth, and for the association between improved inhibitory and attentional control and weight loss [13, 36, 81, 82]. Since these types of training are relatively easy and inexpensive, they might be a feasible and cost-effective way of enhancing existing obesity treatments as early as possible in the life of obese individuals.

In this WELCOME project, a computerized inhibition and attention training in obese children on top of inpatient or outpatient MOT is expected to lead to higher weight loss, in comparison to an active control group. The aim is to reach clinically significant and sustainable weight loss in the experimental group, in line with previous research with comparable self-control trainings on inhibition and attention in obese adults and children. In overweight adults, inhibition trainings with a comparable Go/No Go task reduced impulsive eating and facilitated weight loss [32, 33]. Although the research in children is rather scarce, this also seems to be the case for younger age groups. Both Folkvord et al. [34] and Verbeken et al. [13] showed that comparable inhibition games also reduced calorie consumption and facilitated weight loss in obese children. Next to inhibition, attention training also shows promising results [38]. For example, obese adults in an experimental Food Attention Control Training Program group (FOOD-ACTP) experienced a reduced attentional bias towards unhealthy food, less diet failures and a decreased weight gain [35]. Obese children in an adapted attention modification program also showed a decreased caloric intake [36] but again the research in this target group needs expansion.

Although these results are promising in improving obesity treatments, it is not clear to what extent these effects last over a longer period. Not many studies included follow-up measurements, but the study of Verbeken already made clear that the effects are not always sufficient for a long-term change. That is why, in this project, the focus is not only on enhancing the EF-abilities, but also on evaluating “boosting” the training to gain more long-lasting effects. That is why (a) this treatment not only comprises an intensive training phase but also provides “booster sessions” after their MOT in their own daily home context, and (b) why not only our parameters are measured pre- and post- the training program, but also over a longer follow-up period of 6 months.

It is therefore that it is not sufficient to enhance existing treatment by adding specialized trainings, but that it is also important to focus on long-term changes in the daily life of the obese child. It is only by investigating long-term effects and their transfer to actual lifestyle behavior, that current treatments can be expanded in a way that is feasible and cost-effective for reducing the individual and societal burden of obesity on a long term.

## Abbreviations

BMI: Body Mass Index
EF: Executive Function
IOTF: International Obesity Task Force
MOT: Multidisciplinary Obesity Treatment
OSAS: Obstructive Sleep Apnea Syndrome
RCT: Randomized Controlled Trial
